# The Efficacy and Adverse Reaction of Bleeding of Clopidogrel plus Aspirin as Compared to Aspirin Alone after Stroke or TIA: A Systematic Review

**DOI:** 10.1371/journal.pone.0065754

**Published:** 2013-06-20

**Authors:** Yan Huang, Man Li, Jian-Yong Li, Min Li, Yuan-Peng Xia, Ling Mao, Bo Hu

**Affiliations:** 1 Department of Neurology, Union Hospital, Tongji Medical College, Huazhong University of Science and Technology, Wuhan, China; 2 Key Laboratory of Neurological Disease, Ministry of Education, Tongji Medical College, Huazhong University of Science and Technology, Wuhan, China; University of Queensland, Australia

## Abstract

**Background and Purpose:**

Given the high risk of stroke after TIA (transient ischemia attack) or stroke and the adverse reaction of bleeding of antiplatelets, we undertook a meta-analysis, reviewed randomized controlled trials (RCTs) comparing aspirin plus clopidogrel with aspirin alone to determine the efficacy and adverse reaction of bleeding of the two protocols in the prevention of stroke.

**Methods:**

We analyzed the incidences of stroke, bleeding and severe bleeding by using fixed-effect model or random-effect model on the basis of the result of heterogeneity test.

**Results:**

Five qualified RCTs satisfied the inclusion criteria. We found that treatment with aspirin plus clopidogrel was associated with lower incidence of stroke (Risk Ratio (RR), 0.66, 95% confidence interval (CI), 0.47 to 0.93), higher incidence of bleeding (RR, 1.75, 95% CI, 1.48 to 2.05) as compared with aspirin-alone treatment. In terms of severe bleeding, no statistical difference existed between them (RR, 2.21, 95% CI, 0.25 to 19.52).

**Conclusion:**

The combined use of aspirin and clopidogrel is more effective than aspirin alone for patients with previous TIA or stroke for the prevention of stroke, with risk of bleeding being higher. No statistical difference was found in severe bleeding between the two treatment protocols.

## Introduction

After a transient ischemic attack (TIA) or stroke, patients are at a high risk of suffering from recurrent stroke, which will aggravate stroke-related neurological deficit and sometimes even lead to death. The secondary stroke prevention is especially important for the people who sustain strokes, since each year virtually 30% of strokes are recurrent [1], [2]. The purpose of secondary stroke prevention is to prevent or lessen the recurrent stroke, minimize the severity of post-stroke disability and reduce post-stroke mortality.

Antiplatelet therapy is a major strategy for preventing recurrent stroke in patients with stroke or TIA, as recommended by some guidelines for the control of stroke, such as those formulated by Chinese Guideline for Stroke, the American Stroke Association, and the American Heart Association [1], [3], [4]. These guidelines suggested aspirin, clopidogrel and the combined use of aspirin and extended-release dipyridamole as acceptable alternatives for initial therapy. Because of the low cost and acceptable adverse-effect profile, aspirin is the most widely used antiplatelet agent for the prevention of recurrent stroke. However, the effect of aspirin is limited because it works only in about 15% of recurrent stroke [5], [6]. Furthermore, patients on the treatment may develop aspirin resistance [7]. A study indicates that the dual therapy with clopidogrel and aspirin was more effective than aspirin alone for the prevention of recurrent stroke after TIA or stroke [8].

Nevertheless, clopidogrel and aspirin, as antiplatelet agents, can cause bleeding, with some bleeding cases being mild or asymptomatical bleeding [9]. Another study showed that, a significant increase in life-threatening bleeding was associated with the use of clopidogrel and aspirin in combination [10].

The objective of this study was to systematically review randomized controlled trials that compared the protocol of aspirin plus clopidogrel with aspirin alone in patients with stroke or TIA to determine the efficacy of these therapies in the prevention of the occurrence of stroke and the adverse reaction of bleeding.

## Methods

### Search Strategy for the Targeted Studies

Multiple Electronic databases, including the Cochrane Central Register of Controlled Trials (CENTRAL) on The Cochrane Library, MEDLINE, PUBMED, WEB OF KNOWLEDGE, BIOSIS PREVIEWS, EMBASE, VIP and CNKI were searched. We looked for literatures which were published before September 2011; no lower date limit was applied. No language restrictions were imposed. The key search words included: “aspirin,” “clopidogrel,” “aspirin and clopidogrel,” “aspirin plus clopidogrel,” “antiplatelet therapy,” “stroke,” “transient ischemic attack,” “cerebral ischemia,” “bleeding,” and “severe bleeding”. We also searched the Cochrane Database of Systematic Reviews by using the terms clopidogrel, aspirin, stroke, stroke prevention and identified the most recent reviews to select those clinical trials that satisfied our inclusion criteria. Furthermore, we performed an extensive manual search, examined the reference lists of all relevant publications to find additional studies meeting our inclusion criteria. Finally, we searched websites for recent or ongoing trials.

### Inclusion Criteria

Both published and unpublished trials were considered in our meta-analysis. The inclusion was based on the criteria: (1) randomized controlled trials; (2) clopidogrel plus aspirin and aspirin monotherapy as the treatment regimes or as two groups of the regimes; (3) the patients having a history of stroke or TIA; (4) the occurrence of stroke, bleeding or severe bleeding as the end-point event. Studies involving either dose of the above-mentioned agents or ways of administration of them were seen as eligible. High-quality studies were defined as those double-blinded researches with assessment of compliance and completeness of follow-up, and had blinded outcome adjudication.

### Exclusion Criteria

The studies that involved other platelet aggregation inhibitors (besides aspirin and clopidogrel) as co-intervention were excluded. Non-controlled trials, observational studies, animal studies, reviews, and trials with no rigorous data were also excluded.

### Data Extraction

We chose outcome measures in accordance with the guidelines formulated by the National Institute of Neurological Disorders and Stroke. Stroke included both ischemic and hemorrhagic stroke. Bleeding included mild, moderate and severe bleeding. Severe bleeding was defined as major or life-threatening bleeding. From the trials identified, we (Yan Huang and Man Li) separately extracted relevant clinical information regarding numbers of patients and the numbers of patients experiencing defined outcome events (stroke, bleeding and severe bleeding). Additional information including year of publication, the number of lost subjects, the blindness, the dose of aspirin and clopidogrel, follow-up duration were also collected. Results were double-checked and arbitrated by the another investigator (Jian-Yong Li).

### Statistical Methods

All statistical analyses were conducted by using the Cochrane Collaboration Review Manager software package (Version 5.1 for Mac). The incidences of stroke, the adverse reactions of bleeding and severe bleeding were separately analyzed. For each study, the RR and 95% CI were calculated. The total RRs for the end-point events of stroke and bleeding were obtained by using the Mantel-Haenszel method under the fixed-effect model, and the RR for the outcome of severe bleeding was based on random-effect model. The heterogeneity of all RRs was tested by χ2 test (Woolf method). *Post hoc* analysis was used only for high-quality studies as defined in previous section.

## Results

### Description of the Studies

**Figure 1:**
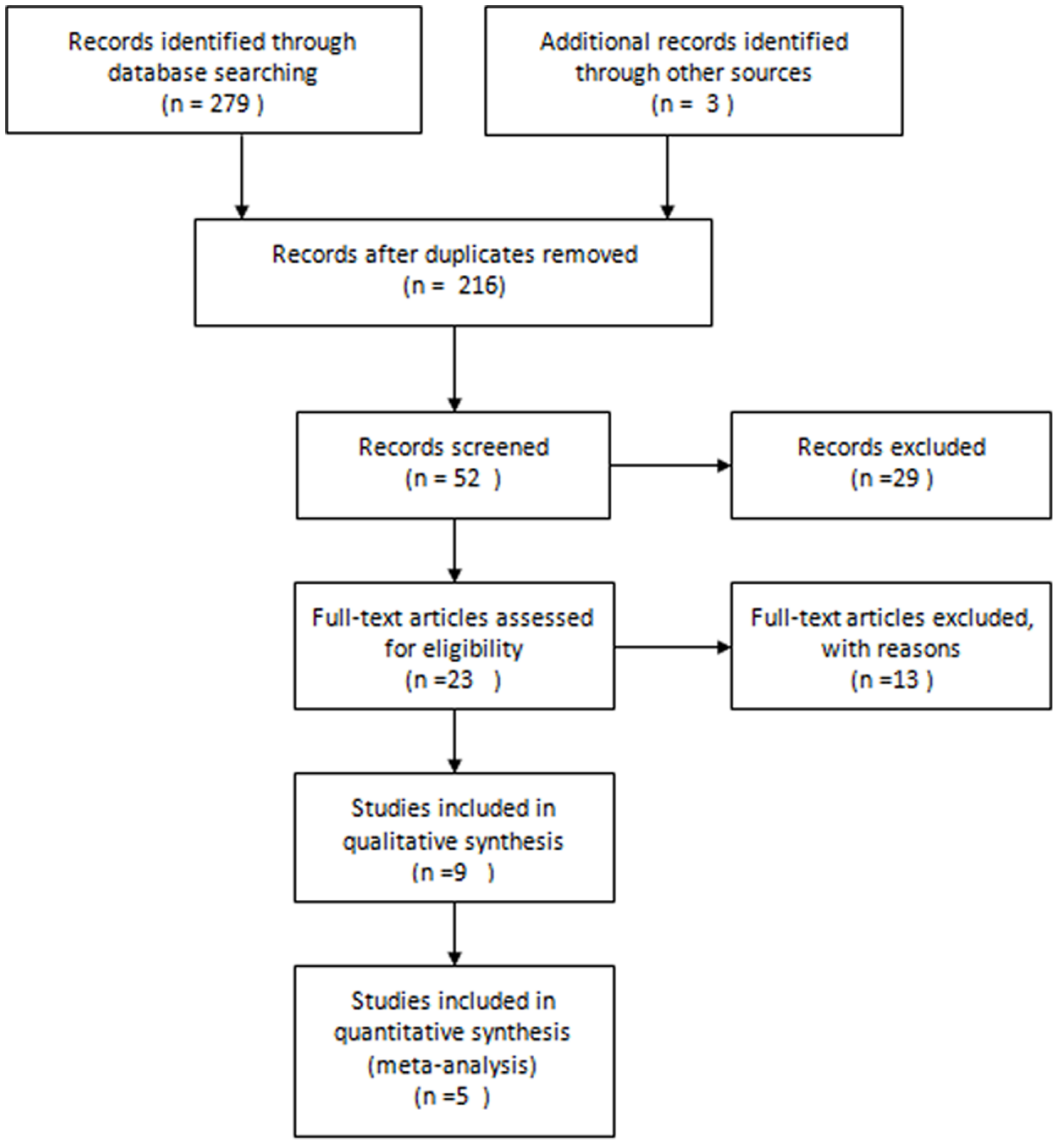
A flow chart showing the progress of trials through the review.

After independent review, Fifty-two trials that compared the combined use of clopidogrel and aspirin with aspirin alone or with aspirin plus placebo in patients with the history of TIA or stroke or those that involved several subgroups including the two aforementioned regimes were considered to be eligible for inclusion in the analysis (Figure 1). Of 52 publications, 29 were excluded because they did not provide acquired data, 13 were excluded because they did not include suitable control groups, 4 were excluded because the same authors published several reports on the same patients. In the end, five clinical trials (involving six papers) were found to meet the inclusion criteria [8], [11]–[15]. The 5 trials included a total of 1950 patients examined for the efficacy of stroke prevention. Among them, 969 patients were allocated to aspirin plus clopidogrel and 981 patients were allocated to aspirin monotherapy or aspirin plus placebo.

In all the five studies (table 1), research was done on the ITT basis and the number of lost subjects during follow-up, though minimal, was recorded. Eventually, 4 studies were identified to satisfy our criteria for high-quality studies.

**Table 1:** Design and Baseline Characteristics of Included Trials.

Study	year of publication	designtype	Size	FU	race/regions	lost subjects	Blinding	patients	Treatment Onset
C. Bal	2009	RCT	22	10 days	Caucasian	0	double-blind	IS/TIA	>8 days
CHARISMA	2011	RCT	1331	25 months	Caucasian, Black, Asian/Oriental	Lost to follow- up _≤0.5%	double-blind	IS/TIA	within 30 days
CLAIR	2010	RCT	98	1 week	Hong Kong, Singapore, China, Thailand, and Malaysia.	1	double-blind	IS/TIA	within 7 days
FASTER	2007	RCT	392	90 days	Canadian	7	double-blind	IS/TIA	within 24 hours
CARESS	2005	RCT	107	1 week	France, Germany, Switzerland, and UK	0	double-blind	IS/TIA	within the last 3 months.

A: aspirin; C: Clopidogrel; A+C: aspirin plus clopidogrel; FU: time of follow-up; IS: ischemic stroke; C. Bal: Effect of the Thromboxane Prostaglandin Receptor Antagonist Terutroban on Arterial Thrombogenesis after Repeated Administration in Patients Treated for the Prevention of Ischemic Stroke; CLAIR:, Clopidogrel plus aspirin versus aspirin alone for reducing embolisation in patients with acute symptomatic cerebral carotid artery stenosis; FASTER: Fast assessment of stroke and transient ischaemic attack to prevent early recurrence; CARESS, Clopidogrel and Aspirin for Reduction of Emboli in Symptomatic Carotid Carotid Stenosis Evaluated Using Doppler Embolic Signal Detection; CHARISMA, The Clopidogrel for High Atherothrombotic Risk and Ischaemic Stabilisation, Management and Avoidance.

The C. Bal study had four groups. We collected data from only two groups that met our requirements. The group of combination regimen consisted of 1 patient with TIA, 9 with infarction (also termed ischemic stroke), and, in the monotherapy group, 2 with TIA, 10 with infarction. Only one patient in the aspirin group suffered from recurrent ischemic stroke. The CLAIR study chose the patients with acute stroke or TIA, stenosis of major arteries and positive microembolic signals (MES). In CHARISMA study, the end-point event of safety included, not limited to, bleeding and severe bleeding. In the CARESS trial, patients positive for MES were randomized to receive dual therapy or monotherapy. There was no episode of serious bleeding in either group. Two papers were about the FASTER study. We could extract the data on incidences of stroke and bleeding from one of the papers ^14^, and obtained the precise data about the incidence of severe bleeding in the other one ^15^. The dosage and administration of antiplatelet agents in these studies are given in table 2.

**Table 2:** Aspirin and Clopidogrel: Total daily dose and the administration.

	Dual therapy (A+C)	Monotherapy (A)
C. Bal	C 75 mg/dayA 300 mg/day	A 300 mg/day
CLAIR	C (300 mg for the first day, then 75 mg daily)A (75–160 mg daily)	A (75–160 mg/day)
CHARISMA	C (75 mg/day) plus A (75–162 mg/day)	A (75–162 mg/day)Placebo
CARESS	C 300-mg on day 1, followed by 75 mg once daily until day 7A 75 mg once daily	A 75 mg once/day daily
FASTER	C(300 mg loading dose then 75 mg daily) for 90 daysA(81 mg, 162 mg if they were naive to aspirin)	A(81 mg, 162 mg if they were naive to aspirin)Placebo

### End Point Stroke for All Studies

**Figure 2:**
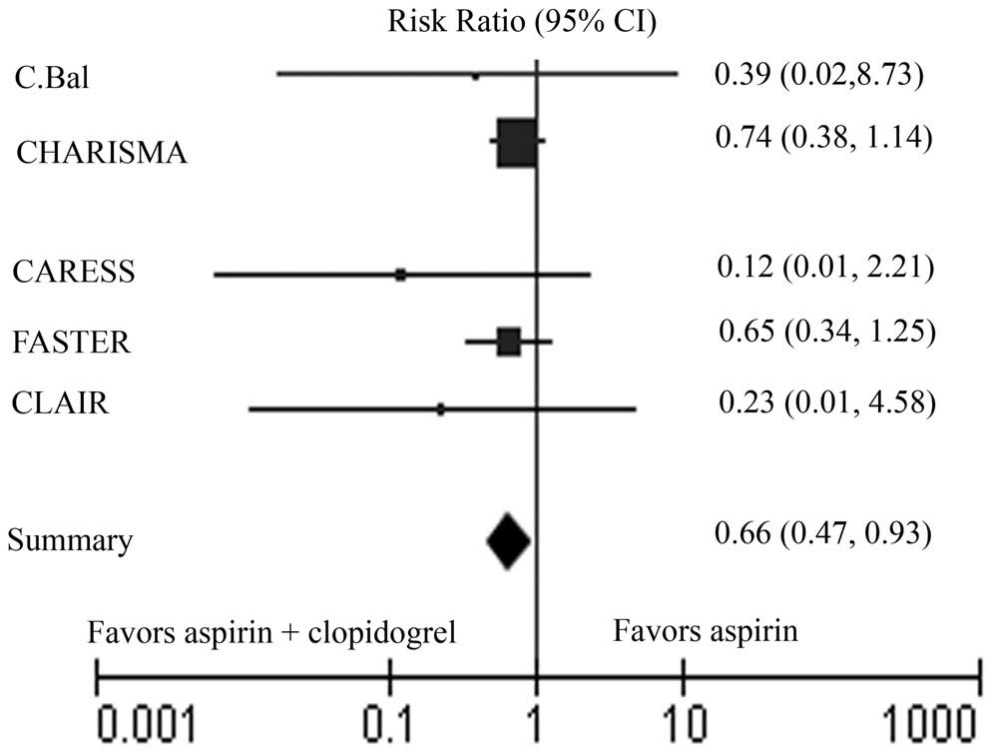
Meta-analysis of all trials comparing clopidogrel plus aspirin with aspirin plus placebo or aspirin alone for stroke. The size of the squares corresponding to each of the trials is inversely proportional to the variance of studies.

Table 3 shows the data of all end-point events in each study. Pertinent data for incidence of stroke were available in all 5 studies. The summary results indicate a significant reduction in the overall RR 0.66 (95% CI 0.47 to 0.93) for clopidogrel combined with aspirin as compared to aspirin alone (Figure 2). The test for heterogeneity of treatment effect across studies revealed no significant difference (Heterogeneity: I^2^ = 0%). The fixed-effect model was used to analyze the data. The incidence of stroke is lower in the dual therapy group.

**Table 3:** Results for end points of stroke-alone, bleeding and severe bleeding.

study	A+C	A	stroke			bleeding			Severe bleeding		
	patients	patients	A+C	A	RR	A+C	A	RR	A+C	A	RR
	(n)	(n)	events	events	95% CI	events	events	95% CI	events	events	95% CI
C. Bal	10	12	0	1	0.39(0.02, 8.73)	0	0		0	0	
CHARISMA	664	667	34	46	0.74(0.48, 1.14)	231	145	1.60(1.34, 1.91)	20	19	1.06(0.57, 1.96)
CLAIR	46	52	0	2	0.23(0.01, 4.58)	2	0	5.64(0.28, 114.49)	0	0	
FASTER	198	194	14	21	0.65(0.34, 1.25)	67	27	2.43(1.63, 3.63)	5	0	10.78 (0.60,193.62)
CARESS	51	56	0	4	0.12(0.01, 2.21)	2	1	2.20(0.21, 23.50)	0	0	

n: number of patients or events in each subgroup; RR: risk ratio.

### Bleeding Data of 6 Studies

**Figure 3:**
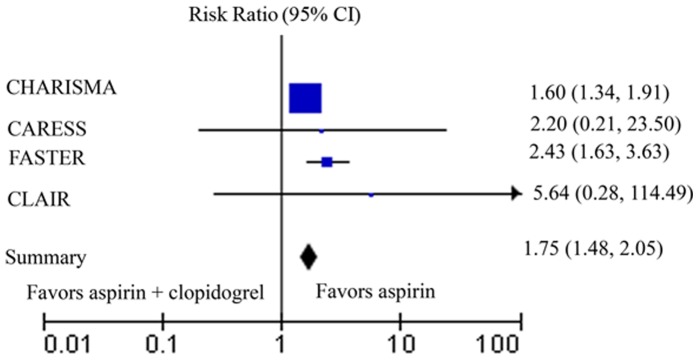
Meta-analysis of all trials comparing clopidogrel plus aspirin with aspirin plus placebo or aspirin alone for the adverse reaction of bleeding.

Outcome data for bleeding were available from 4 studies. In the C. Bal dit study, no patients developed bleeding, including severe bleeding, in the two groups we targeted. The test for heterogeneity of adverse reaction-bleeding across studies exhibited no significant difference in the incidence of bleeding (Heterogeneity: I^2^ = 0%) (Figure 3). We also used Mantel-Haenszel method under the fixed-effect model to process bleeding data. The RR for the event of bleeding was 1.75 (95% CI 1.48 to 2.05). The incidence of bleeding was significantly higher in the clopidogrel-plus-aspirin group than aspirin-alone group.

### Severe Bleeding as the End Point

**Figure 4:**
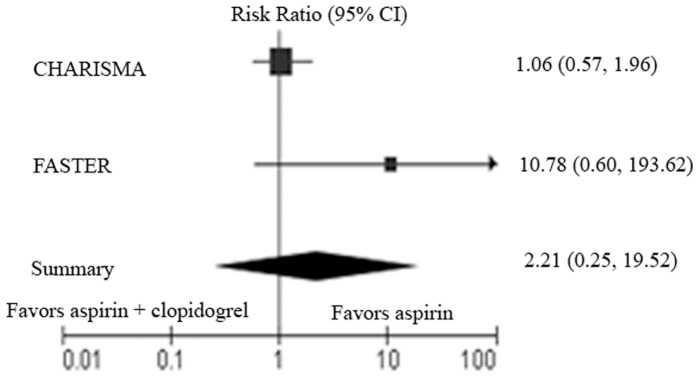
Meta-analysis of all trials comparing clopidogrel plus aspirin with aspirin plus placebo or aspirin alone for adverse events of severe bleeding.

No severe bleeding was reported in three of the five studies. We collected the data of severe bleeding in the other two reports and subjected them to a meta-analysis (Figure 4). The test for heterogeneity of severe bleeding across studies showed significant differences (Heterogeneity: I^2^ = 60%). Our analysis with the random-effect model showed that the sum RR was 2.21 (95% CI, 0.25 to 19.52). No statistical difference was found between the dual therapy group and the monotherapy group.

## Discussion

The results of this meta-analysis show that the combination of aspirin plus clopidogrel is more effective than aspirin alone in preventing stroke in patients with previous stroke or TIA, with increased risk of bleeding spontaneously. But the difference between the two groups is not significant in severe bleeding. When data from all 5 original trials are pooled, the results show a statistically significant benefit in favor of aspirin plus clopidogrel compared with aspirin monotherapy for the stroke-alone end point. According to the outcome of bleeding, the risk of dual therapy is higher compared with the monotherapy. About the severe bleeding end point, the variance is not clear between the two protocols. Our meta-analysis pooled data from high quality studies as well as those having a potential for bias because of weaknesses in quantity of patients and study design: however, when our analysis was limited to the 4 studies considered high quality and the studies with more participants separately, the results were essentially the same. It illustrates that bias introduced by the studies with less participants and the lower quality studies could not explain the results of our meta-analysis.

In the trials followed-up for short time, C Bal dit (10 days), CLAIR (7 days), CARESS (7 days), there is no stroke and severe bleeding about the dual therapy group. In the monotherapy group, it is 5.83 percentage about the outcome of stroke. Then it is safe and more effective in the acute phase–the period of high risk of stroke after TIA or stroke according to outcomes of stroke and severe bleeding to use the regime of clopidogrel plus aspirin for short time.

Some comments are necessary for a correct interpretation of these data. In the C. Bal dit study, the outcomes of bleeding and severe bleeding were not available in the published papers straightforward, so we inferred them from the paper after we affirmed that they could not be obtained from the trial investigators. In the CHARISMA study, the severe bleeding included severe and moderate bleeding.

Table 4 shows the characteristics of patients in each studies included. Some characteristics are different in statistically between the two groups. The spaces participated in studies were not the same, and the proportion of Asian is low. So we need more similar trials adjusted our Asian to verify the outcomes of the two protocols. There are differences in the time from onset of stroke or TIA to randomization which differs from 24 hours to 3 months. And we know that we should use antiplatelet agents as soon as possible to reduce stroke recurrence. In the age of patients participated, we can find the variance too. Most of all, the previous diseases of the patients are not the same in every study too.

**Table 4:** Patients’ characteristics for included trials.

Trial	Trt.	N	Male	Age* (Y)	Prior MI (%)	Prior PVD (%)	Prior angina (%)	HT (%)	HL (%)	DM (%)
CHARISMA	A+C	664	393 (59.2%)	66.1		25 (3.8)		477 (71.8)		191 (28.8)
	A	667	414 (62.1%)	66.3		25 (3.7)		489 (73.3)		174 (26.1)
FASTER	A+C	198	113 (57.1%)	70.2						
	A	194	94 (48.5%)	70.0						
CARESS	A+C	51	35 (68.6%)	66.4	6 (11.8)	11 (21.6)	11 (21.6)	38 (74.5)	28 (54.9)	16 (31.4)
	A	56	39 (69.6%)	62.8	10 (17.9)	6 (10.7)	13 (23.2)	31 (55.4)	32 (57.1)	18 (32.1)
C. Bal	A+C	10	8 (80.0%)	67.8						
	A	12	8 (66.7%)	70.5						
CLAIR	A+C	46	36 (78.3%)	59.2	3 (6.5)	4 (8.7)	5 (10.9)	27 (58.7)	23 (50)	21 (45.7)
	A	52	40 (76.9%)	56.4	3 (5.8)	2 (3.8)	4 (7.7)	35 (67.3)	16 (30.8)	16 (30.8)

N: number of patients or events in each subgroup; Trt: treatment; MI: myocardial infarction; PVD: peripheral vascular disease; HT: Hypertension; HL: Hyperlipidaemia; DM: diabetes mellitus; d: days; m: months; w: weeks. *Mean.

The SPS-3 trial is also a RCT comparing the two protocols. The results are not included because the trial has not been published, and we can’t obtain the data we want. The study was stopped prematurely because of increased bleeding events and poor outcomes and without any evidence of efficacy in preventing recurrent stroke. This trial, which is comparable in size to CHARISMA, may result in bias in our meta-analysis.

The Chamila M. study [16] compared the safety and efficacy of dual versus mono antiplatelet therapy. There are two different combinations of dual antiplatelet therapy–aspirin plus clopidogrel and aspirin plus dipyridamole–with 3 different single antiplatelets–aspirin, clopidogrel, and dipyridamole. The patients with acute (≤3 days) ischemic stroke/TIA were enrolled in the included trials. On one hand, though the conclusion of this meta-analysis included more aspects such as composite vascular events, the overall sample size about aspirin plus clopidogrel vs. aspirin was small. On the other hand, most of the publications did not give all the necessary data and the authors had to contact the investigators for additional data. Bias might result due to the missing data.

In summary, our meta-analysis of aspirin plus clopidogrel compared to aspirin alone or aspirin plus placebo for the prevention of stroke and the risk of bleeding after stroke or TIA shows a robust benefit, with higher risk of bleeding with the treatment, but the difference of risk of severe bleeding was not statistically significant for the combination use compared to aspirin alone. Considered the differences among the original trials prescribed previously, although the severe bleeding between the dual therapy and the monotherapy is not very different according to the forest plots, there is an upward tendency in the dual therapy group. Nevertheless, we can’t conclude that the benefit/risk ratio is worthy using the combination use of clopidogrel and aspirin, because the safety outcome should include death and other vascular diseases. We need more exact clinical trials to refine the best suited regimes for the stroke prevention after stroke or TIA.

## Supporting Information

Table S1 PRISMA 2009 CHECKLIST.
(DOC)
